# Pulsed field ablation of spatiotemporal electrogram dispersion following pulmonary vein isolation and left atrial linear lesions for persistent atrial fibrillation: a case report

**DOI:** 10.1093/ehjcr/ytae085

**Published:** 2024-02-09

**Authors:** Vasileios Sousonis, Quentin Voglimacci-Stephanopoli, Sarah Zeriouh, Serge Boveda, Jean Paul Albenque

**Affiliations:** Heart Rhythm Management Department, Clinique Pasteur, 45 Avenue de Lombez, 31300 Toulouse, France; Heart Rhythm Management Department, Clinique Pasteur, 45 Avenue de Lombez, 31300 Toulouse, France; Heart Rhythm Management Department, Clinique Pasteur, 45 Avenue de Lombez, 31300 Toulouse, France; Heart Rhythm Management Department, Clinique Pasteur, 45 Avenue de Lombez, 31300 Toulouse, France; Heart Rhythm Management Department, Clinique Pasteur, 45 Avenue de Lombez, 31300 Toulouse, France

**Keywords:** Persistent atrial fibrillation, Pulsed field ablation, Electroporation, Catheter ablation, Electrogram dispersion, Case report

## Abstract

**Background:**

Ablation of persistent atrial fibrillation (AF) remains challenging, with atrial substrate modification often being performed as an adjunct to pulmonary vein isolation (PVI). Pulsed field ablation (PFA) is a novel ablation modality that carries a favourable safety profile, which could facilitate complex procedures.

**Case summary:**

We present the case of a 60-year-old male undergoing catheter ablation for symptomatic persistent AF. The procedure was performed with the Farapulse™ PFA system in a stepwise manner, including PVI and linear lesions for the isolation of the posterior left atrial wall and the ablation of the mitral isthmus. The final step of the procedure included the ablation of areas exhibiting spatiotemporal electrogram dispersion, identified with the help of artificial intelligence–based software (VX1, Volta Medical) in both atria. Sinus rhythm was restored after the abolition of an electrogram dispersion zone in the right atrium. The procedure was carried out without any complications.

**Discussion:**

Complex ablation procedures for persistent AF can be successfully performed with PFA. In the context of such extensive ablation strategies, PFA is an attractive energy source, given its non-thermal nature that is known to prevent damage to surrounding tissue and result in less chronic fibrosis. However, caution should be exercised to avoid excessive ablation when using the currently available multispline PFA catheter, as it may inadvertently target adjacent areas of healthy myocardium.

**Learning points**

- An ablation procedure for persistent atrial fibrillation (AF), combining pulmonary vein isolation with posterior wall isolation, ablation of the mitral isthmus, and abolition of dispersion electrograms, can be successfully performed with the Farapulse™ pulsed field ablation (PFA) system.
- Given its favourable safety profile, PFA is an attractive alternative to thermal energy sources for complex ablation procedures for persistent AF, as it prevents damage to surrounding structures.

## Introduction

Despite pulmonary vein isolation (PVI) being a fundamental part of persistent atrial fibrillation (AF) ablation, the complex pathophysiology of this arrhythmia often necessitates additional substrate modification. Therefore, electrogram-guided ablation has been used as an adjunct to PVI, yielding encouraging results in recent studies.^1,2^ Recently, artificial intelligence (AI)–based software applications were developed, facilitating the identification and ablation of areas characterized by spatiotemporal electrogram dispersion, which are believed to correspond to the electrical footprints of AF drivers.^3,4^

Pulsed field ablation (PFA) is a non-thermal ablation modality that employs short, high-amplitude electric pulses to induce cardiac cell death. The arrhythmia-free survival following PFA for AF appears to be comparable to that of thermal ablation.^5,6^ In addition, owing to its favourable safety profile,^7^ the clinical application of PFA has rapidly expanded beyond PVI.^8^

We present a case of PFA for persistent AF, performed in a stepwise procedure involving PVI, linear lesions, and AI-guided ablation of dispersion electrograms.

## Summary figure

**Figure:**
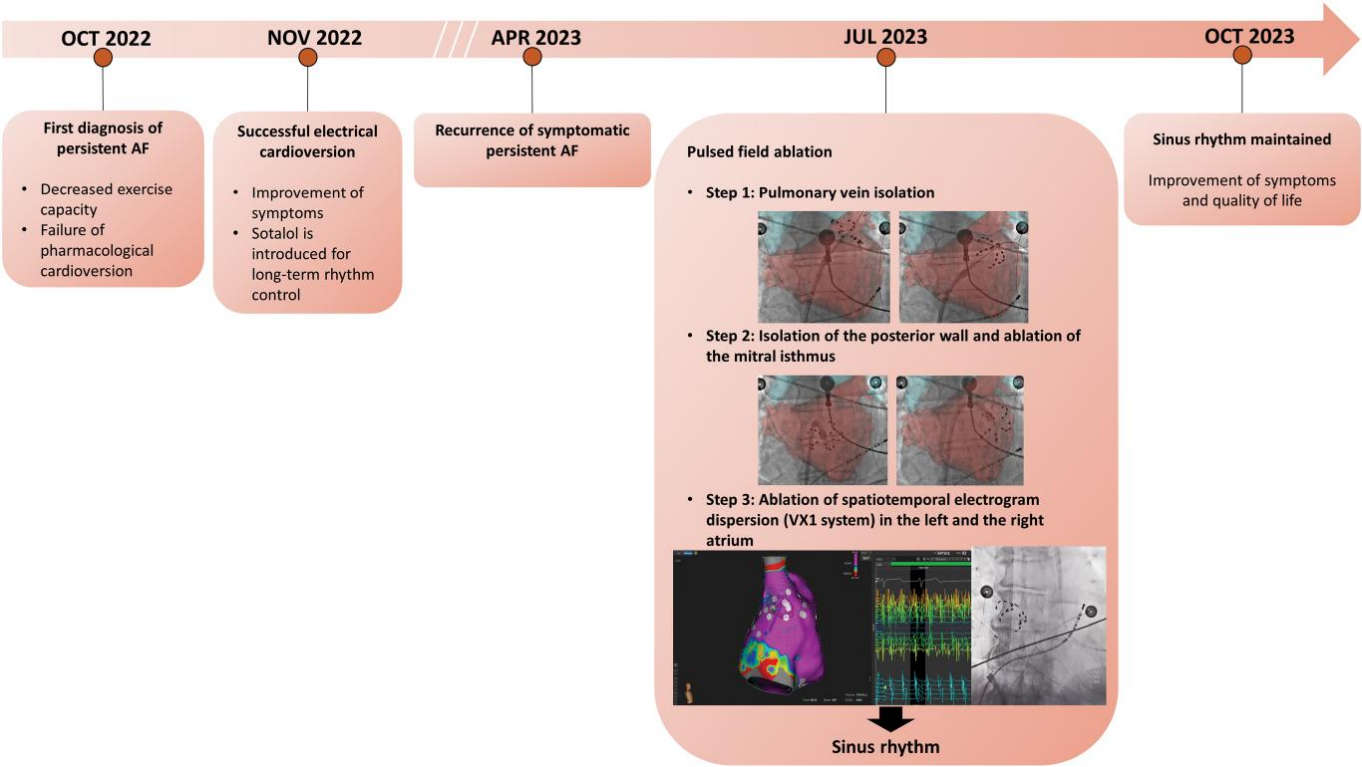


## Case report

A 60-year-old male, with a medical history of arterial hypertension and persistent AF, was assessed in our department. Despite having undergone a prior electrical cardioversion and being treated with sotalol, the patient complained of limited exercise capacity, in the context of a recurring episode of persistent AF. As a result, catheter ablation was proposed. The potential risks and benefits were thoroughly discussed with the patient, who consented to an experimental ablation procedure using PFA for PVI, linear lesions, and AI-guided ablation of spatiotemporal dispersion zones.

**Figure 1:**
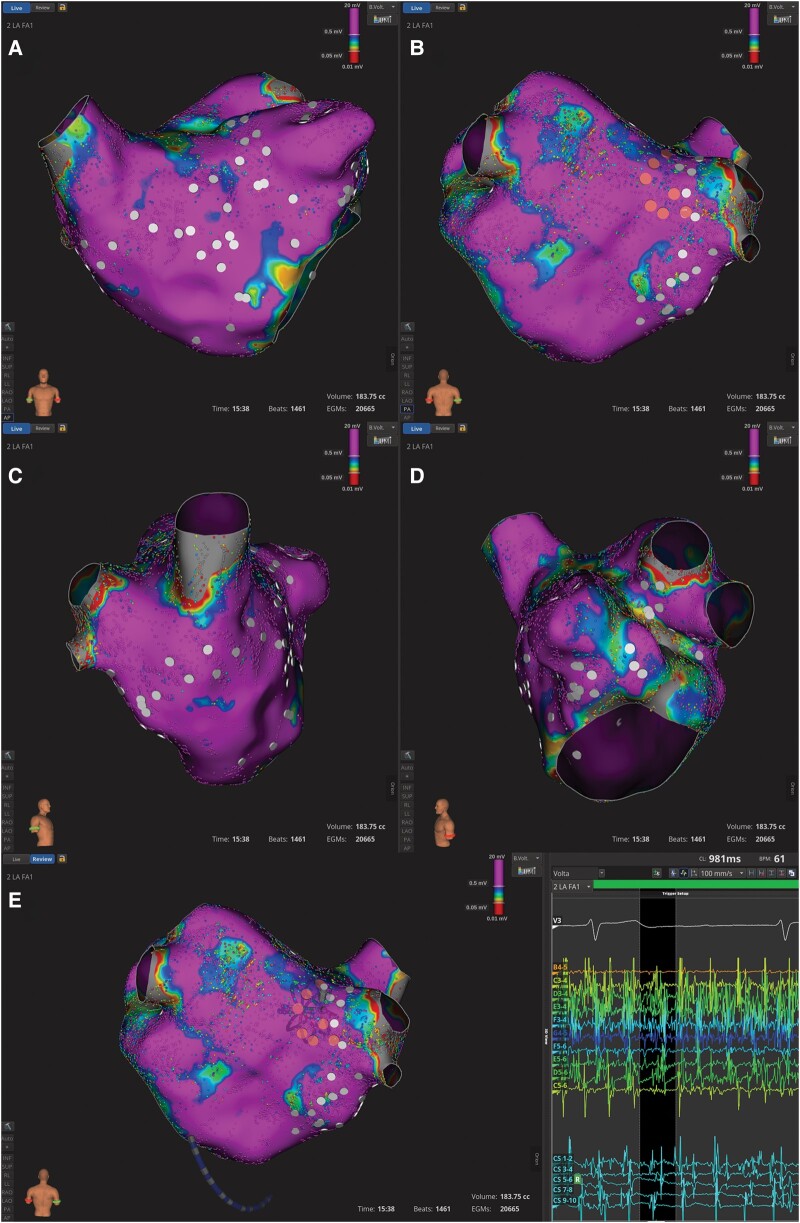
3D electroanatomic voltage map of the left atrium in atrial fibrillation. Tags represent electrogram dispersion zones (orange tags: zones of high spatiotemporal dispersion; white tags: zones of moderate spatiotemporal dispersion). (*A*) Anterior view. (*B*) Posterior view. (*C*) Right lateral view. (*D*) Left lateral view. (*E*) Electrograms exhibiting spatiotemporal dispersion around the right pulmonary vein antrum.

**Figure 2:**
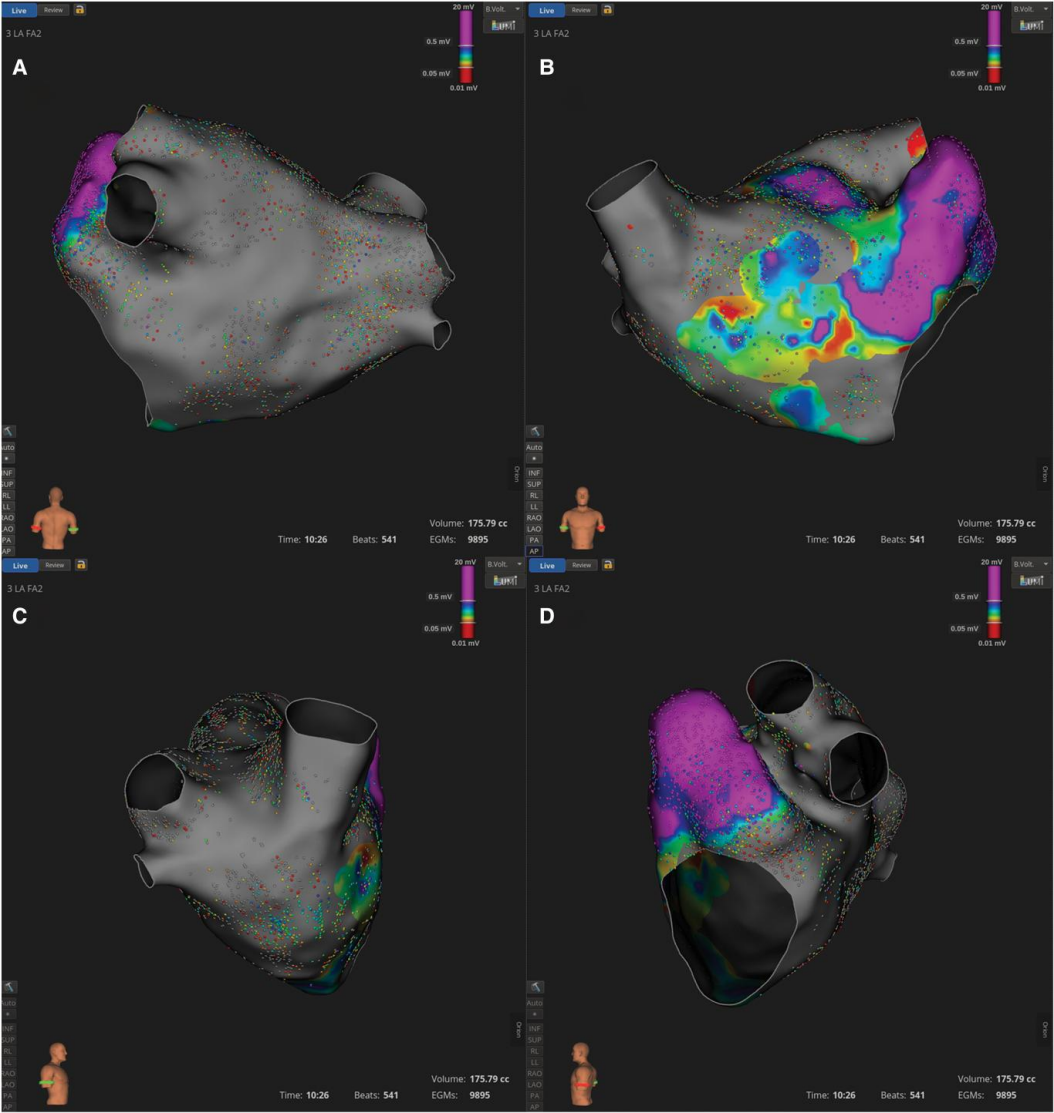
3D electroanatomic voltage map of the left atrium following pulmonary vein isolation, isolation of the posterior wall, and ablation of the mitral isthmus and zones of spatiotemporal electrogram dispersion. (*A*) Posterior view. (*B*) Anterior view. (*C*) Right lateral view. (*D*) Left lateral view.

**Figure 3:**
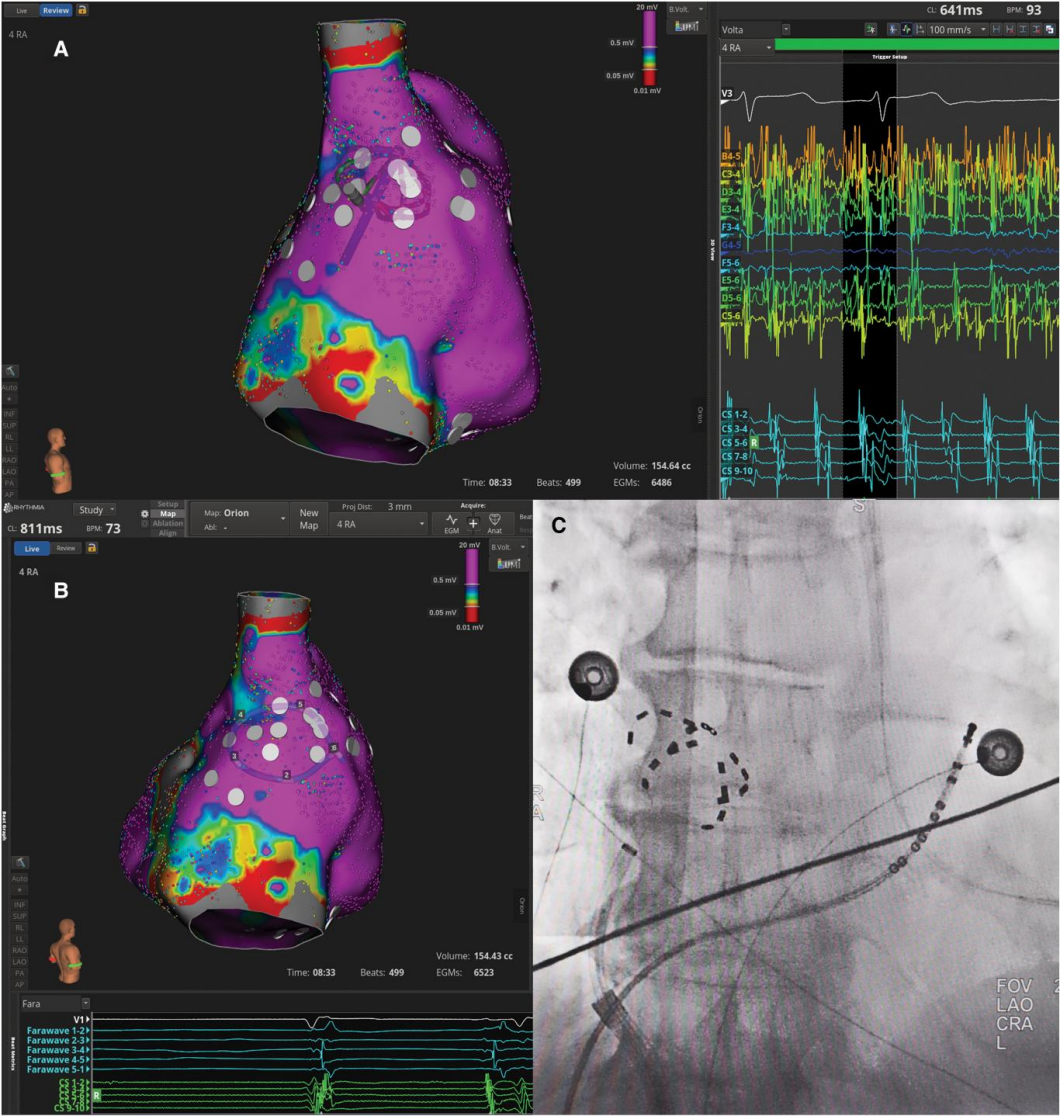
Ablation of dispersion zones in the right atrium. (*A*) Left: 3D electroanatomic voltage map of the right atrium in atrial fibrillation. White tags represent moderate dispersion zones. Right: electrograms showing spatiotemporal dispersion on the lateral wall of the right atrium. (*B*) Site of atrial fibrillation termination after pulsed field ablation of dispersion electrograms. The multispline pulsed field ablation catheter is visualized as a circular catheter on the 3D electroanatomic map of the right atrium. (*C*) Fluoroscopy image of the position of the multispline pulsed field ablation catheter in the right atrium during the coupled energy applications that led to atrial fibrillation termination, in postero-anterior projection.

The procedure was performed under general anaesthesia. A preoperative computed tomography scan revealed a dilated left atrium (LA) of 60 mL/m^2^ and confirmed the absence of thrombi. The ablation was performed in AF, using the Farapulse™ PFA system (Boston Scientific, USA). Following femoral venous access, a steerable decapolar catheter (Inquiry™, St Jude, USA) was placed into the coronary sinus, and a single transseptal puncture was performed under fluoroscopy guidance, using a Swartz™ SL0 sheath (Abbot, USA) and a BRK™ XS needle (St Jude, USA). High-density 3D electroanatomic mapping was performed with an eight-spline multipolar catheter (Intellamap Orion™, Boston Scientific, USA) and the Rhythmia HDx™ mapping system (Boston Scientific, USA). Areas with electrograms exhibiting spatiotemporal dispersion were identified in real time with the VX1 software (Volta Medical, France) and were manually annotated on the 3D electroanatomic map. Dispersion zones were found around the pulmonary vein antra, at the ostium of the LA appendage, and on the anterior LA wall and the septum (*Figure 1*). Next, the sheath was exchanged for a deflectable Faradrive™ sheath (Boston Scientific, USA). Under fluoroscopy guidance, PVI was performed with a 35 mm multispline PFA catheter (Farawave™, Boston Scientific, USA). Based on its impedance characteristics, the latter could be visualized as a circular catheter on the 3D electroanatomic map, further facilitating its positioning. Eight applications were administered per vein (two pairs in the basket, followed by two pairs in the flower configuration, with a 45° catheter rotation between each pair). Each PFA application comprised a train of five consecutive pulses of 2.0 kV for a total duration of 2.5 s. The next step included the isolation of the posterior LA wall and the creation of a posterior mitral line by linear PFA lesions, as previously described.^8^ Next, we targeted every dispersion zone in the LA that had not been affected during the previous steps, ensuring the preservation of the appendage (*Figure 2*). Since the sinus rhythm was not restored, the right atrium was mapped for dispersion electrograms. Notably, dispersion clusters were identified on the lateral wall of the right atrium and along the cavotricuspid isthmus (*Figure 3A*). Atrial fibrillation was successfully terminated after the first pair of PFA applications on the lateral wall of the right atrium (*Figure 3B* and *C*; Supplementary material online, *Video S1*). Direct visualization of the Farawave™ catheter as a circular catheter on the 3D electroanatomic map allowed us to precisely assess its position in relation to the junction of the superior vena cava and the right atrium before energy application, preventing any unintentional damage to the sinus node. Following the restoration of sinus rhythm, the isolation of the pulmonary veins and the posterior wall was verified, and a bidirectional block across the mitral isthmus was confirmed by differential pacing. The procedure was carried out without any complications.

At 3-month follow-up, the patient was in sinus rhythm and had a marked improvement in his symptoms and quality of life.

## Discussion

Pulsed field ablation is a highly promising ablation modality with increasing clinical applications. We present the first case of AI-guided PFA of dispersion electrograms in both atria, performed as the final step of an ablation procedure for persistent AF, following PVI and linear LA lesions.

Substrate ablation is often performed for non-paroxysmal AF. Given that extensive ablation close to surrounding structures is often required, these procedures pose a significant risk of collateral damage. Pulsed field ablation emerges as an attractive alternative to radiofrequency, as its cardioselectivity prevents damage to surrounding tissue, while simultaneously ensuring lesion transmurality.^7,9^ In the presented case, we were able to safely ablate the posterior LA wall and the lateral wall of the right atrium, which are in close relation to the oesophagus and the right phrenic nerve, respectively.

So far, PFA has been used in complex ablation procedures for persistent AF, which combine PVI with posterior wall isolation and linear ablation of the mitral isthmus, with encouraging short-term outcomes.^8^ Additional electrogram-guided ablation has been associated with improved arrhythmia-free survival^1,3^ and was performed as a final step in the presented case. This approach facilitated the restoration of sinus rhythm by targeting dispersion electrograms in the right atrium. Although there is some controversy in the literature concerning the role of procedural AF termination as an endpoint for the ablation of non-paroxysmal AF,^10,11^ a recent study indicated that, when dispersion electrograms detected with the VX1 system are targeted, procedural AF termination is associated with improved long-term outcomes.^3^ However, when PFA is used, determining whether AF termination results from the ablation of a site critical for AF maintenance or from local electrical cardioversion due to the ultra-rapid electrical field might not be straightforward.

A potential drawback of targeting complex atrial electrograms is the lack of specificity.^12^ This can lead to extensive ablation, resulting in pro-arrhythmic zones of electrical heterogeneity while, at the same time, raising significant considerations regarding the long-term mechanical function of the LA. Pulsed field ablation could mitigate these concerns to some extent, as it is known to produce more uniform lesions compared with radiofrequency,^13^ with minimal complex electrical activity at the lesion borders.^14^ Additionally, PFA is associated with less chronic fibrosis and improved regional mechanics compared with thermal ablation.^15^ However, the use of the multispline PFA catheter to target small zones of spatiotemporal dispersion could result in inadvertent ablation of areas not participating in the pathogenesis of AF. Whether the ablation of such regions could interfere with LA mechanics or even lead to a higher thromboembolic risk in the case of extensive applications merits further investigation. The aforementioned concerns, along with the unintentional isolation of the left atrial appendage and the occurrence of conduction disturbances when areas close to the conduction system are targeted, should be acknowledged as potential risks of the described procedure, and caution should be taken when utilizing the multispline PFA catheter to target complex atrial electrograms.

In conclusion, we present an ablation strategy for persistent AF, combining PVI with posterior wall isolation, ablation of the mitral isthmus, and bi-atrial ablation of dispersion electrograms with PFA. Pulsed field ablation presents potential advantages over radiofrequency in such procedures. However, caution should be exercised to avoid excessive ablation using the currently available technology, and solid procedural endpoints should be established. The introduction of newer-generation PFA catheters, designed for more precise energy application, is expected to further facilitate these complex procedures.

## Supplementary Material

ytae085_Supplementary_Data

## Data Availability

The data underlying this article are available in the article and its online supplementary material.

## Lead author biography

Dr Vasileios Sousonis is a fellow in cardiac electrophysiology in Clinique Pasteur, Toulouse, France. His main area of interest is catheter ablation of cardiac arrhythmias and cardiac pacing.

## Supplementary material

**Consent:** A written informed consent, in line with COPE guidelines, was obtained from the patient for the publication of his clinical information and imaging data.

**Funding:** None declared.
